# Variation at 2q35 (*PNKD* and *TMBIM1*) influences colorectal cancer risk and identifies a pleiotropic effect with inflammatory bowel disease

**DOI:** 10.1093/hmg/ddw087

**Published:** 2016-03-22

**Authors:** Giulia Orlando, Philip J. Law, Kimmo Palin, Sari Tuupanen, Alexandra Gylfe, Ulrika A. Hänninen, Tatiana Cajuso, Tomas Tanskanen, Johanna Kondelin, Eevi Kaasinen, Antti-Pekka Sarin, Jaakko Kaprio, Johan G. Eriksson, Harri Rissanen, Paul Knekt, Eero Pukkala, Pekka Jousilahti, Veikko Salomaa, Samuli Ripatti, Aarno Palotie, Heikki Järvinen, Laura Renkonen-Sinisalo, Anna Lepistö, Jan Böhm, Jukka-Pekka Mecklin, Nada A. Al-Tassan, Claire Palles, Lynn Martin, Ella Barclay, Albert Tenesa, Susan Farrington, Maria N. Timofeeva, Brian F. Meyer, Salma M. Wakil, Harry Campbell, Christopher G. Smith, Shelley Idziaszczyk, Timothy S. Maughan, Richard Kaplan, Rachel Kerr, David Kerr, Daniel D. Buchanan, Aung Ko Win, John Hopper, Mark Jenkins, Noralane M. Lindor, Polly A. Newcomb, Steve Gallinger, David Conti, Fred Schumacher, Graham Casey, Jussi Taipale, Jeremy P. Cheadle, Malcolm G. Dunlop, Ian P. Tomlinson, Lauri A. Aaltonen, Richard S. Houlston

**Affiliations:** ^1^Division of Genetics and Epidemiology, The Institute of Cancer Research, London SW7 3RP, UK; ^2^Genome-Scale Biology Research Program, Research Programs Unit; ^3^Department of Medical and Clinical Genetics, Medicum; ^4^Institute for Molecular Medicine Finland, University of Helsinki, Helsinki 00014, Finland; ^5^National Institute for Health and Welfare, Helsinki 00271, Finland; ^6^Folkhälsan Research Centre, Helsinki 00250, Finland; ^7^Unit of General Practice and Primary Health Care, University of Helsinki and Helsinki University Hospital, Helsinki 00014, Finland; ^8^Finnish Cancer Registry, Institute for Statistical and Epidemiological Cancer Research, Helsinki 00130, Finland; ^9^School of Health Sciences, University of Tampere, Tampere 33014, Finland; ^10^Analytic and Translational Genetics Unit, Department of Medicine, Massachusetts General Hospital, Boston, MA 02114, USA; ^11^Program in Medical and Population Genetics, The Broad Institute of MIT and Harvard, Cambridge, MA 02142, USA; ^12^Department of Neurology, Massachusetts General Hospital, Boston, MA 02114, USA; ^13^Department of Surgery, Helsinki University Central Hospital, Hospital District of Helsinki and Uusimaa, Helsinki 00029, Finland; ^14^Department of Surgery, Abdominal Center, Helsinki University Hospital, Helsinki 00029, Finland; ^15^Department of Pathology, Central Finland Central Hospital, Jyväskylä 40620, Finland; ^16^Department of Surgery, Jyväskylä Central Hospital, University of Eastern Finland, Jyväskylä 40620, Finland; ^17^Department of Genetics, King Faisal Specialist Hospital and Research Center, Riyadh 12713, Saudi Arabia; ^18^Wellcome Trust Centre for Human Genetics and NIHR Comprehensive Biomedical Research Centre, Oxford OX3 7BN, UK; ^19^Colon Cancer Genetics Group, University of Edinburgh and MRC Human Genetics Unit, Western General Hospital, Edinburgh EH4 2XU, UK; ^20^The Roslin Institute, University of Edinburgh, Easter Bush, Roslin EH25 9RG, UK; ^21^Centre for Population Health Sciences, University of Edinburgh, Edinburgh EH8 9AG, UK; ^22^Institute of Cancer and Genetics, School of Medicine, Cardiff University, Cardiff CF14 4XN, UK; ^23^CRUK/MRC Oxford Institute for Radiation Oncology, University of Oxford, Oxford OX3 7DQ, UK; ^24^MRC Clinical Trials Unit, Aviation House, London WC2B 6NH, UK; ^25^Department of Oncology, Oxford Cancer Centre, Churchill Hospital; ^26^Nuffield Department of Clinical Laboratory Sciences, John Radcliffe Hospital, University of Oxford, Oxford OX3 7LE, UK; ^27^Colorectal Oncogenomics Group, Genetic Epidemiology Laboratory, Department of Pathology; ^28^Centre for Epidemiology and Biostatistics, The University of Melbourne, Melbourne, Vic. 3010, Australia; ^29^Department of Health Sciences Research, Mayo Clinic, Scottsdale, AZ 85259, USA; ^30^Cancer Prevention Program, Fred Hutchinson Cancer Research Center, Seattle, WA 98109, USA; ^31^Lunenfeld-Tanenbaum Research Institute, Mount Sinai Hospital, Toronto, ON M5G 1X5, Canada; ^32^Department of Preventive Medicine, University of Southern California, Los Angeles, CA 90033, USA; ^33^Department of Biosciences and Nutrition, SciLife Center, Karolinska Institute, Stockholm, SE 141 83, Sweden

## Abstract

To identify new risk loci for colorectal cancer (CRC), we conducted a meta-analysis of seven genome-wide association studies (GWAS) with independent replication, totalling 13 656 CRC cases and 21 667 controls of European ancestry. The combined analysis identified a new risk association for CRC at 2q35 marked by rs992157 (*P* = 3.15 × 10^−8^, odds ratio = 1.10, 95% confidence interval = 1.06–1.13), which is intronic to *PNKD* (paroxysmal non-kinesigenic dyskinesia) and *TMBIM1* (transmembrane BAX inhibitor motif containing 1). Intriguingly this susceptibility single-nucleotide polymorphism (SNP) is in strong linkage disequilibrium (*r*^2 ^= 0.90, *D*′ = 0.96) with the previously discovered GWAS SNP rs2382817 for inflammatory bowel disease (IBD). Following on from this observation we examined for pleiotropy, or shared genetic susceptibility, between CRC and the 200 established IBD risk loci, identifying an additional 11 significant associations (false discovery rate [FDR]) < 0.05). Our findings provide further insight into the biological basis of inherited genetic susceptibility to CRC, and identify risk factors that may influence the development of both CRC and IBD.

## Introduction

Colorectal cancer (CRC), a leading cause of cancer-related death worldwide, has a heritable basis (1,2). Recent genome-wide association studies (GWAS) have successfully identified a number of common single-nucleotide polymorphisms (SNPs) influencing CRC risk thereby vindicating the assertion that part of the heritable risk is polygenic (3–7). These studies have also provided insights into the biology of CRC, highlighting the importance of bone morphogenetic protein signalling pathway genes (*BMP2, BMP4, GREM1* and *SMAD7*) (4,5), candidate genes (*CDH1*), as well as genes not previously implicated in CRC (*POLD3, TERC, CDKN1A, VIT1A* and *SHROOM2*) (6,7). It is well established that inflammatory bowel disease (IBD), which primarily presents as Crohn’s disease or ulcerative colitis, is associated with an increased CRC risk (8–11). Despite IBD being strongly heritable (12), little evidence for shared genetic susceptibility or differential effects of genetic variation on IBD and CRC risk has been reported, although the presumption is that the direction of effect will be consistent between both diseases.

A failure to uncover pleiotropy may be reflective of a lack of power of CRC GWAS conducted thus far. Indeed statistical modelling of GWAS data shows that although 19% of the heritability of CRC can be ascribed to common variation, only 10% of this is explained by currently identified risk SNPs (13). To empower the identification of new CRC susceptibility SNPs in persons of European ancestry, we conducted a genome-wide meta-analysis of a previously unreported GWAS with six published datasets in addition to independent replication totalling 13 810 cases and 21 754 controls.

We report the identification of a new CRC risk association which also impacts on IBD risk. Extending our analysis to established IBD loci, we provide evidence of shared genetic susceptibility between CRC and IBD at 11 additional loci.

## Results

### Primary GWAS

In the primary scan (termed the FIN GWAS), 1172 CRC cases ascertained through the Finnish CRC collection and Finnish Cancer Registry were analysed with control data on 8266 individuals from the FINRISK, Health2000, Finnish Twin Cohort and Helsinki Birth Cohort Study cohorts. After applying strict quality control criteria, 283 906 autosomal SNPs were available for association with CRC risk. A quantile–quantile (Q–Q) plot of observed versus expected χ^2^-test statistics showed little evidence for an inflation of test statistics, thereby excluding the possibility of substantive hidden population substructure, cryptic relatedness among subjects or differential genotype calling (inflation factor *λ* = 1.07).

### Meta-analysis

We performed a meta-analysis of our primary scan data with six other non-overlapping GWAS of European ancestry (CCFR1, CCFR2, COIN, UK1, Scotland1 and VQ58), which have been previously reported (14). To maximize the prospects of identifying novel risk variants, we imputed the data with a merged reference panel using Sequencing Initiative Suomi (SISu) (for the FIN data) or UK10K (for the UK data) in addition to 1000 Genomes Project data. After quality control procedures, over 10 million variants, including over 1 million insertion–deletions, were analysed in 8749 cases and 18 245 controls.

Associations for the 37 previously established European CRC risk SNPs showed a direction of effect consistent with previously reported studies, with 10 of these SNPs having *P* < 5.0 × 10^−^^8^ in this meta-analysis (Supplementary Material, Table S1). Excluding these known risk SNPs, together with those correlated with *r*^2^ > 0.8, from the meta-analysis two novel regions of linkage disequilibrium (LD), marked by rs992157 and rs2383207, showed the strongest association with CRC at *P *< 1.0 × 10^−^^6^ (Supplementary Material, Table S2).

**Figure 1.**
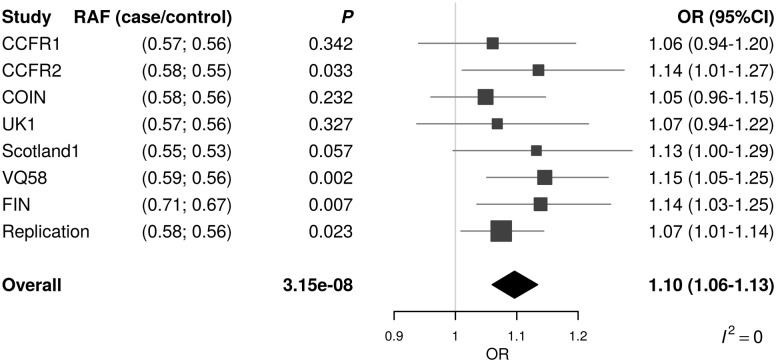
Forest plot of the odds ratios for the association between rs992157 and CRC. Studies were weighted according to the inverse of the variance of the log of the OR. Horizontal lines: 95% confidence intervals (95% CI)*.* Box: OR point estimate; its area is proportional to the weight of the study. Diamond: overall summary estimate, with confidence interval given by its width. Vertical line: null value (OR = 1.0).

**Figure 2.**
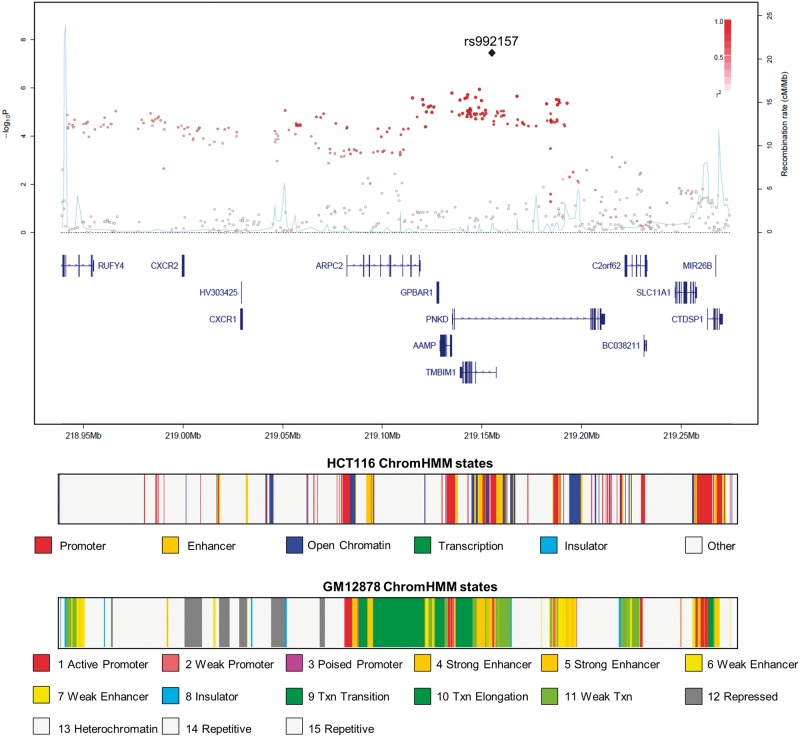
Regional plot of association results and recombination rates for the 2q35 locus. In the panel, −log_10_ *P* values (*y*-axis) of the SNPs are shown according to their chromosomal positions (*x*-axis). The top SNP is shown as a large triangle and is labelled by its rsID. The colour intensity of each symbol reflects the extent of LD with the top SNP: white (*r*^2 ^= 0) through to dark red (*r*^2 ^= 1.0), with *r*^2^ estimated from the 1000 Genomes Phase 1 data. Genetic recombination rates (cM/Mb) are shown with a light blue line. Physical positions are based on NCBI build 37 of the human genome. Also shown are the relative positions of genes and transcripts mapping to each region of association. The lower panel shows the chromatin state segmentation track (ChromHMM) in HCT116 CRC and GM12878 lymphoblastoid cell lines.

To replicate these associations, we genotyped rs992157 and rs2383207 in an additional 5061 CRC cases and 3509 controls, with only rs992157 showing evidence for an association with CRC (*P *= 0.023). In the combined analysis, the association was significant at the genome-wide threshold (*P *= 3.15 × 10^−^^8^; Fig. 1). There was no variation due to heterogeneity (*I*^2^ = 0, *P*_het_ = 0.79). rs992157 is located at 2q35, and is intronic to two genes: paroxysmal non-kinesigenic dyskinesia (*PNKD*) on the forward strand and transmembrane BAX inhibitor motif containing 1 (*TMBIM1*) on the reverse strand (Fig. 2).

### Relationship between genotype and CRC phenotype

Using data on microsatellite instability (MSI) status from the FIN (*n* = 1146), COIN (*n* = 1239) and NSCCG replication (*n* = 1282) series, together with information on KRAS and BRAF mutation status in tumours in COIN, we explored the possibility that the association at rs992157 is restricted to a specific molecular subtype of CRC (Supplementary Material, Table S3). There was no evidence of an association between these SNPs and any of the variables after adjusting for multiple testing (i.e. *P* > 0.05). Additionally, we observed no consistent association between age, sex or tumour site using data from the UK1, Scotland1, VQ58, COIN and NSCCG series (Supplementary Material, Table S3).

### IBD SNPs influence CRC

**Figure 3.**
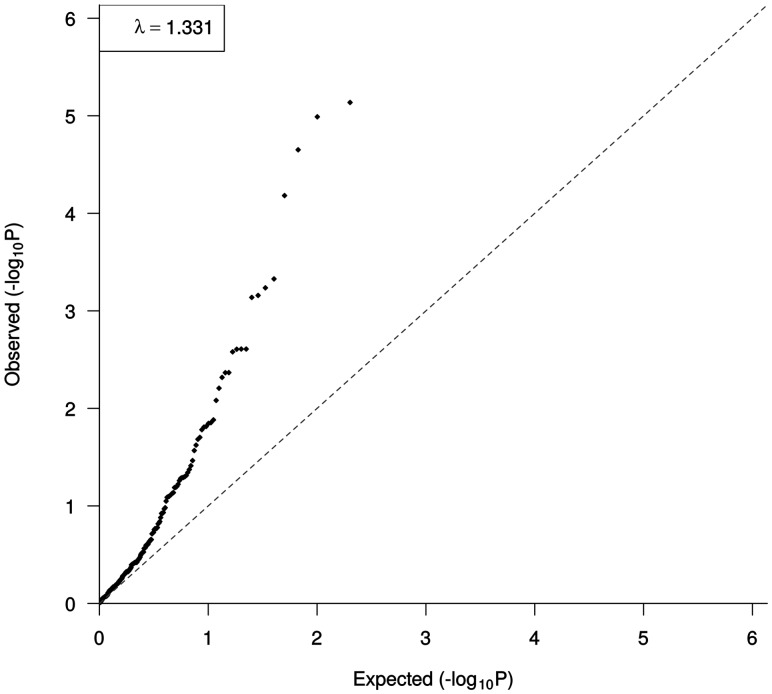
Quantile–quantile (Q–Q) plot of observed and expected CRC association *P*-values for 200 IBD risk SNPs (15, 16).

Another association at 2q35 defined by rs2382817 has previously been shown to influence IBD risk (CRC meta *P* = 1.02 × 10^−^^5^), which is also intronic to *PNKD* and *TMBIM1*, and is in strong LD with rs992157 (*r*^2 ^= 0.90, *D*′ = 0.96). Paradoxically, the risk for rs2382817 in IBD is inverse to the CRC association. Given the compelling evidence for an association between IBD and CRC, we sought evidence for additional shared susceptibility between the two diseases. Specifically, we examined the risk of CRC in our meta-analysis at 200 loci that have been shown in previous GWAS to affect IBD risk (15,16) (Supplementary Material, Table S4). A Q–Q plot of the observed CRC association *P*-values against the expected *P*-values for each of the 200 IBD risk SNPs showed significant over-dispersion (*λ* = 1.33, Fig. 3). This observation is compatible with a genetic relationship between CRC and IBD.

To account for multiple testing, we imposed an FDR-adjusted *P*-value of 0.05 as being statistically significant. At this threshold, in addition to rs2382817, 11 IBD risk SNPs were associated with CRC risk (Table 1), of which five were positively associated with CRC risk, whereas the other seven displayed an inverse relationship. A number of these SNPs annotate genes with documented roles that are relevant to CRC development, such as Wnt-signalling [*WNT4*, (17)], tumour suppression [*MAPKAPK5*, *FOXO1* (18,19)] and cellular transformation [*CDC42*, *CEBPB* (20,21)] (Table 1). We examined for an association between the genotype of these 12 SNPs and the molecular subtype of CRC, and found no evidence of a relationship (Supplementary Material, Table S3).

**Table 1.** Table of the IBD SNPs with FDR-corrected *P*-value <0.05 in the CRC GWAS

rsID	Chr	Position	Tag genes	CRC risk allele	IBD risk allele	CRC RAF	CRC *P*-value	CRC FDR corrected	CRC OR	CRC 95% CI
rs12568930	1	22702231	WNT4, CDC42	T	T	0.85	6.58 × 10^−05^	3.29 × 10^−03^	1.12	(1.06; 1.18)
rs7554511	1	200877562	GPR25, C1orf106	A	C	0.29	6.95 × 10^−04^	0.02	1.08	(1.03; 1.13)
rs7608910	2	61204856	PUS10, REL	A	G	0.63	7.28 × 10^−04^	0.02	1.07	(1.03; 1.12)
rs17229285	2	199523122	PLCL1, SATB2	C	C	0.49	2.46 × 10^−03^	0.04	1.06	(1.02; 1.1)
rs2382817	2	219151218	TMBIM1, PNKD	C	A	0.62	1.02 × 10^−05^	1.02 × 10^−03^	1.09	(1.05; 1.14)
rs4722672	7	27231762	HOXA13, HOXA11	C	C	0.20	2.46 × 10^−03^	0.04	1.08	(1.03; 1.13)
rs174537	11	61552680	MYRF, TMEM258	G	T	0.67	2.63 × 10^−03^	0.04	1.06	(1.02; 1.11)
rs653178	12	112007756	ATXN2, MAPKAPK5	T	C	0.54	2.23 × 10^−05^	1.49 × 10^−03^	1.09	(1.05; 1.13)
rs17085007	13	27531267	GPR12, UPS12	C	C	0.19	5.81 × 10^−04^	0.02	1.09	(1.04; 1.15)
rs941823	13	41013977	MRPS31, FOXO1	T	C	0.27	2.47 × 10^−03^	0.04	1.07	(1.02; 1.12)
rs516246	19	49206172	FUT2, MAMSTR	T	T	0.54	4.71 × 10^−04^	0.02	1.07	(1.03; 1.11)
rs913678	20	48955424	CEBPB, PTPN1	C	T	0.34	7.30 × 10^−06^	1.02 × 10^−03^	1.10	(1.05; 1.14)

### Functional effect prediction analysis

The genomic region containing rs992157 is the site of active structure and has regulatory motifs for both enhancer and promotor function in multiple cell types (Fig. 2). Moreover ChIP-seq data identify over 122 transcription factors binding to the region, including CRC-related transcription factors such as MYC, HNF4A and TCF7L2 (Supplementary Material, Table S5). We also performed an expression quantitative trait loci (eQTL) analysis and found no significant relationship between the rs992157 genotype and *PNKD* and *TMBIM1* expression in colorectal adenocarcinoma cells (Supplementary Material, Table S6). The risk genotype was however associated with altered gene expression in other tissues, including lymphoblastoid cells (FDR *P*-value < 0.05, Supplementary Material, Table S6). This apparent difference in eQTLs may be reflective of the differences in epigenetic profiles at 2q35 between CRC and lymphoblastoid cells (Fig. 2).

To further investigate the relationship between CRC and IBD risk we performed eQTL analysis on the 12 IBD SNPs associated with CRC risk in the colorectal adenocarcinoma data, and found two significant relationships between rs174537 and the expression of fatty acid desaturase 2 (*FADS2*, FDR *P*-value = 3.28 × 10^−^^6^) and between rs516246 and fucosyltransferase 2 (*FUT2*, FDR *P*-value = 2.08 × 10^−^^17^) (Supplementary Material, Table S6). Additional evidence for these eQTLs was found in other tissues in the Geuvadis, Blood and GTEx databases (Supplementary Material, Table S6). Similarly to rs992157, as reported above, rs2382817 is an eQTL for *PNKD* and *TMBIM1* in both lymphoblastoid and whole blood tissues.

**Figure 4.**
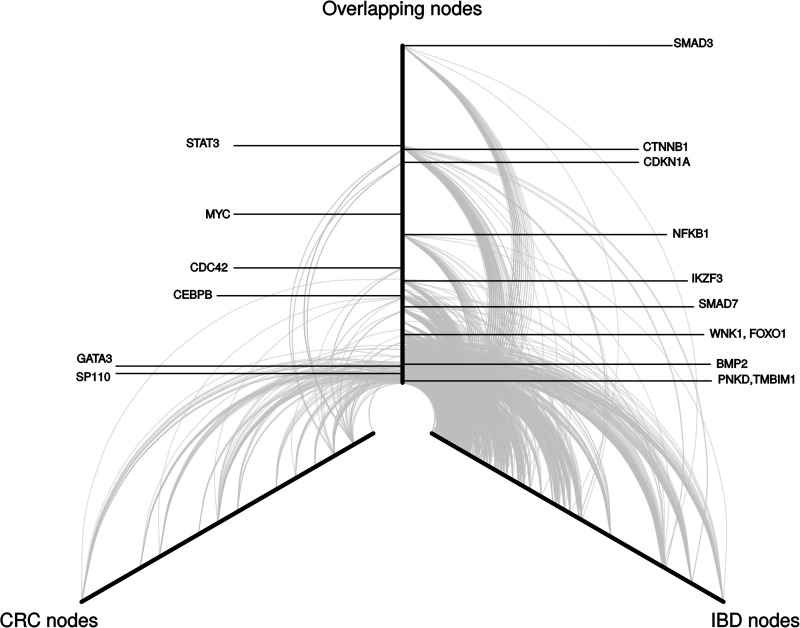
Hive plot of common protein–protein interactions between CRC and IBD defined by risk SNPs. Each arc represents an interaction between two proteins, and the distance from the centre of the plot corresponds to a greater number of protein–protein interactions (higher degree of the node). The left arm represents proteins that were only identified using the CRC SNPs, the right arm represents proteins that were only identified using the IBD SNPs, and the central arm represents the common proteins, highlighting the previously associated tag genes.

Following on from this we investigated the presence of shared genetic pathways between CRC and IBD using the LENS pathway tool (22), which allows exploration of interactions between the gene products in proximity to the GWAS SNPs. Across the 594 CRC proteins and 1574 IBD proteins, a network of 542 overlapping proteins was identified. Figure 4 shows the common network and interactions between key proteins. Of interest was the direction of association between the CRC SNPs with IBD risk. Pathways with evidence of enrichment (i.e. *P *< 0.001) with a consistent effect between CRC and IBD were involved in immune and inflammatory response, such as co-stimulation by the CD28 family, Fc epsilon receptor signalling and downstream B-cell receptor signalling. In contrast, the protein networks defined by reciprocal SNPs association for CRC and IBD were enriched for interleukin and calmodulin signalling. Pathways that were enriched in both, albeit involving different proteins, included those related to the adaptive immune response, cytokine signalling and interferon signalling (Supplementary Material, Table S7).

## Discussion

In this meta-analysis we combined seven independent GWAS, and have identified a risk locus for CRC risk at 2q35 marked by rs992157. As this SNP is intronic to both *PNKD* and *TMBIM1*, and these are the only transcripts within the region of high LD, it is a plausible that the genetic basis of the 2q35 association for CRC is through functional effects on one of these *genes* a priori. This is coupled with the fact that rs992157 localizes to a genomic region with regulatory function and the eQTL data showing allele-specific *cis*-regulatory relationship between SNP genotype and *PNKD* and *TMBIM1* expression. Although speculative, the long isoform of *PNKD* appears to function in a pathway to detoxify alpha-ketoaldehyde using glutathione as a cofactor (23). As glutathione is essential for maintaining cellular redox status, reduced glutathione levels in cells through dysfunctional PNKD may lead to increasing oxidative stress levels, which have been linked to inflammation (24). TMBIM1 has been reported to have a role in regulating the level of Fas ligand (25,26), which mediates both apoptosis and inflammation (27). Therefore, both gene products indirectly contribute to the regulation of inflammation, a physiological process linked with the onset of IBD and CRC.

Another SNP in the 2q35 locus (rs2382817), which is in strong LD with rs992157, has previously been shown to influence IBD risk (15). In addition, contemporaneous with our analysis, a recent study (28) has also found evidence, albeit not GWAS significant, for a relationship between 2q35 variation and CRC risk (*P *= 7.0 × 10^−^^5^), additionally finding an inverse relationship with risk of IBD. The identified SNP, rs11676348, is correlated with both rs992157 and rs2382817 (LD metrics, *r*^2^ and *D*′ = 0.32, 0.65 and 0.33, 0.71, respectively). The opposing effects of the rs2382817-C allele with increased risk of CRC but decreased risk of IBD may initially appear paradoxical, given the increased risk of CRC associated with IBD. The risk of CRC in IBD increases with longer duration, extent of colitis and the degree of inflammation (11). The inflammatory response has been linked to increased oxidative stress, and this oxidative state stimulates antioxidant defences that promote the survival pathways in cancer cells, favouring tumour proliferation (29). Nonetheless, these SNPs may indicate shared pathways in which there are opposing relationships between carcinogenesis and inflammation.

Motivated by the observation that the 2q35 locus influences IBD risk, we sought additional evidence for a common genetic basis for both diseases by evaluating the CRC risk at previously established IBD loci (15,16). While not formally significant globally, there was an over-representation of association signals for CRC defined by the IBD risk SNPs. Through this analysis we identified potential risk variants for CRC mapped to regions in the proximity of genes encoding WNT4 and CDC42, previously shown to be involved in the risk of CRC (14); MAPKAPK5, a member of the MAPK family reported to regulate MYC protein levels (18); and the transcription factor CEBPB, found to be highly expressed in samples derived from CRC patients (21). Moreover, our eQTL analysis on IBD SNPs showed altered expression of *FADS2* and *FUT2* genes in CRC tissues. Both the genes have previously been reported to have a role in the development of IBD (30,31) providing further evidence of possible shared genes. Further studies are required to delineate the genetic basis and implicate perturbation of a specific gene as the functional basis of the associations. Collectively these data are consistent with a degree of commonality in genetically defined pathways in the development between CRC and IBD, albeit that many of the associations have opposite effects.

Considering the low prevalence of IBD in European populations (<0.5%) (32), together with the observation that other SNPs that are strongly associated with risk of IBD were not associated with CRC, it is unlikely that sampling has biased our findings. Moreover if the association between these IBD SNPs and CRC was simply mediated by its association with IBD *per se*, we would have expected directionality of the association to be identical but this was not the case for many of the SNPs.

In summary, we have identified a new risk association for CRC which also influences IBD risk. Our association signals for CRC defined by other established IBD risk SNPs also serve to highlight the importance of shared gene pathways in the development of CRC and IBD. Deciphering the functional and biological basis of these SNPs associations has the potential to translate into a better understanding of the biological basis of how IBD transitions to CRC. Finally our analysis serves to illustrate that inter-relationships between diseases do not necessarily equate to consistent allelic architecture in risk, thus adding an extra layer of complexity to interpretation.

## Materials and Methods

### Ethics

Collection of blood samples and clinico-pathological information from subjects was undertaken with informed consent and ethical review board approval at all sites in accordance with the tenets of the Declaration of Helsinki.

### Primary GWAS

The Finnish GWAS (FIN) was based on 1172 CRC cases and 8266 cancer free controls ascertained through Finnish Hospitals (33) and through the Finnish Cancer Registry. Cases were genotyped using Illumina HumanOmni 2.5M8v1 according to the manufacturer’s recommendations. For controls, we made use of Illumina HumanHap 670k and 610k array data on individuals from the FINRISK (34), Health 2000 (35), Finnish Twin Cohort (36) and Helsinki Birth Cohort Studies (37). Individuals were excluded with: <90% successfully genotyped SNPs, discordant sex information, duplication or cryptic relatedness (identity by descent > 0.2). We excluded SNPs from the analysis with: call rate < 95%, (minor allele frequency [MAF]) < 0.01 and departure from Hardy–Weinberg equilibrium in controls at *P* < 10^−^^6^. The adequacy of the case–control matching and the possibility of differential genotyping of cases and controls were assessed using quantile–quantile (Q–Q) plots of test statistics.

### Published GWAS for meta-analysis

We made use of six previously published GWAS: UK1 (CORGI study) (7) comprised 940 cases with colorectal neoplasia and 965 controls; Scotland1 (COGS study) (7) included 1012 CRC cases and 1012 controls; VQ58 comprised 1800 CRC cases from the UK-based VICTOR and QUASAR2 adjuvant chemotherapy clinical trials (38) and 2690 population control genotypes from the Wellcome Trust Case Control Consortium 2 (WTCCC2) 1958 birth cohort (39); CCFR1 comprised 1290 familial CRC cases and 1055 controls from the Colon Cancer Family Registry (CCFR) (40); CCFR2 included a further 796 cases from the CCFR and 2236 controls from the Cancer Genetic Markers of Susceptibility (CGEMS) studies of breast and prostate cancer (41,42); and the COIN GWAS (14) was based on 2244 CRC cases ascertained through two independent Medical Research Council clinical trials of advanced/metastatic CRC (COIN and COIN-B) (43) and controls comprised 2162 individuals from the UK Blood Service Control Group genotyped as part of the WTCCC2 (39).

The VQ58, UK1 and Scotland1 GWAS series were genotyped using Illumina Hap300, Hap240S, Hap370, Hap550 or Omni2.5M arrays. 1958BC genotyping was performed as part of the WTCCC2 study on Hap1.2M-Duo Custom arrays. The CCFR samples were genotyped using Illumina Hap1M, Hap1M-Duo or Omni-express arrays. CGEMS samples were genotyped using Illumina Hap300 and Hap240 or Hap550 arrays. The COIN cases were genotyped using Affymetrix Axiom Arrays and the Blood Service controls were genotyped using Affymetrix 6.0 arrays. After applying the same quality control as that performed for FIN, data on 8749 CRC cases and 18 245 controls were available for the meta-analysis.

The adequacy of the case–control matching and possibility of differential genotyping of cases and controls were assessed using Q–Q plots of test statistics. *λ*_GC_ values (44) for the UK1, Scotland1, VQ58, CCFR1, CCFR2 and COIN studies were 1.02, 1.01, 1.01, 1.02, 1.03 and 1.05, respectively. Any ethnic outliers or individuals identified as related were excluded.

### Replication series

In total, 5061 CRC cases from the National Study of Colorectal Cancer Genetics (NSCCG) (45) were genotyped. Controls (*n* = 3509) were from NSCCG and the Genetic Lung Cancer Predisposition Study (46). None of the controls had a known history of malignancy at ascertainment. All subjects were British residents with self-reported European ethnicity and there were no obvious demographic differences between cases and controls. DNA was extracted from EDTA-venous blood samples using conventional methodologies and PicoGreen quantified (Invitrogen Corporation, Carlsbad, CA, USA). Genotyping of two SNPs was conducted using KASPar competitive allele-specific PCR chemistry (LGC, Hoddesdon, UK; primer sequences and conditions available on request). To monitor quality control, duplicate samples were included in assays, and concordance between duplicate samples was >99%.

### Imputation and meta-analysis

Analyses were undertaken using R (v3.02) (47) and PLINK (v1.9) (48) software. Phasing of GWAS SNP genotypes was performed using SHAPEIT (v2.r644 and v2.r790 for FIN) (49). Prediction of the untyped SNPs was carried out using IMPUTE (v2.3.1) (50). The FIN dataset used a merged reference panel based on data from the 1000 Genomes Project (Phase 1 v3) (51) together with an additional population matched reference panel of 3882 SISu haplotypes. The UK samples used a merged reference panel using data from the 1000 Genomes Project and UK10K (April 2014 release). The fidelity of imputation, as assessed by the concordance between imputed and sequenced SNPs, was examined in a subset of 200 UK cases (14). The association between each SNP and the risk of CRC was assessed by a frequentist association test under an additive model, using SNPTEST (v2.5.1) (52), utilizing the genotype probabilities from IMPUTE where an SNP was not directly typed. Population stratification was controlled in the FIN samples using sex and six principal components. Association meta-analyses only included markers with info scores >0.8, imputed call rates/SNP >0.9 and MAFs >0.005. Meta-analyses were carried out using META (v1.6) (53). We calculated Cochran’s *Q* statistic to test for heterogeneity and the *I*^2^ statistic to quantify the proportion of the total variation that was caused by heterogeneity (54). *I*^2^ values ≥75% are considered characteristic of large heterogeneity (54).

### Characterization of cancer phenotype

Associations by sex, age and clinico-pathological phenotypes were examined by logistic regression. MSI status was determined using BAT25 and BAT26 markers, and samples showing ≥5 novel alleles when compared with normal DNA at either or both markers were assigned as MSI-H (corresponding to MSI-high) (55). Tumours were screened for *KRAS* codons 12, 13 and 61 and *BRAF* codon 600 mutations by pyrosequencing (43). Additionally, *KRAS* (all three codons) and *BRAF* (codons 594 and 600) were screened for mutations by MALDI-TOF mass array (Sequenom, San Diego, CA, USA) (56). Differences between the various sites of the tumour (colonic [ICD-9:153], rectal [ICD-9:154.1] and recto sigmoid junction [ICD9:154.0]) were also analysed.

### Functional prediction

To explore epigenetic profiles of genomic location associated with CRC, we used ENCODE histone modification data, HaploReg and RegulomeDB (57,58) to examine whether any of the SNPs or their proxies (i.e. *r*^2 ^> 0.8 in the 1000 Genomes EUR reference panel) annotate transcription factor binding or enhancer elements. Additionally, we made use of ChIP-seq data on the LoVo CRC cell line (59). We used ChromHMM to integrate DNase, H3K4me3, H3K4me1, H3K27ac, Pol2 and CTCF states from the CRC cell line HCT116 using a multivariate Hidden Markov Model (60). ChromHMM tracks for lymphoblastoid cells were obtained from ENCODE (61). We assessed sequence conservation using: PhastCons (62) (>0.3 indicative of conservation) and Genomic Evolutionary Rate Profiling (63) (>2 indicative of conservation). SNAP plots were created using the visPIG tool (64).

### eQTL analysis

To examine for a relationship between SNP genotype and mRNA expression in CRC, we analysed Tumor Cancer Genome Atlas (TCGA) RNA-seq expression and Affymetrix 6.0 SNP data (dbGaP accession number: phs000178.v7.p6) on 416 colorectal adenocarcinoma samples (65). Association between normalized RNA counts per-gene and SNP genotype was quantified using the Kruskal–Wallis trend test. To look for a relationship between SNP genotype and expression levels in other tissues, we used publicly available expression data generated from the MuTHER (66), eQTL Blood Browser (67), GTEx (68) and Geuvadis/1000 Genomes (69) resources. For the Geuvadis data, the relationship between SNPs and expression of genes located within 1 Mb was analysed using the Matrix eQTL (70) package under a linear model. When the SNPs were not directly typed, a proxy SNP was used (*r*^2 ^≥ 0.8). In all the datasets, eQTL results were included where FDR *P *< 0.05.

### Relationship between established risk SNPs for IBD and CRC

To investigate pleiotropic (shared genetic susceptibility) between CRC and IBD, we examined the 201 SNPs identified in GWAS that have been shown to affect IBD risk (15,16). One SNP (rs71559680) is an indel that was not present in the CRC genotyping arrays or the reference panels, and was thus removed from the analysis. We obtained the lead SNPs from the IBD GWAS and extracted the *P*-values for the corresponding SNPs in our CRC meta-analysis.

### Pathway analysis

To investigate the possibility of shared genetic susceptibility between CRC and IBD, we performed pathway analysis. First, we selected the two closest coding genes for the leading SNPs in each GWAS and then performed pathway analysis using LENS tool (22), which identifies gene product and protein–protein interactions from HPRD (71) and BioGRID (72). Enrichment of pathways was assessed using Fisher’s exact test, comparing the overlap of the genes in the network with the genes in the pathway. Pathway data were obtained from REACTOME (73). Cytoscape was used to perform network analyses (74), and the Hive Plot was drawn using HiveR (academic.depauw.edu/∼hanson/HiveR/HiveR.html, last accessed March 29, 2016).

## Supplementary Material

Supplementary Material is available at *HMG* online.

Supplementary Data
