# Supplementary material for: Variation at 2q35 (PNKD and TMBIM1) influences colorectal cancer risk and identifies a pleiotropic effect with inflammatory bowel disease
Source: Hum Mol Genet. 2016 Mar 22;25(11):2349–59. doi: 10.1093/hmg/ddw087 (PMC5081051; doi:10.1093/hmg/ddw087)
Supplement: Supplementary Data [file supp_25_11_2349__index.html]

Variation at 2q35 (PNKD and TMBIM1) influences colorectal cancer risk and identifies a pleiotropic effect with inflammatory bowel disease — Variation at 2q35 (PNKD and TMBIM1) influences colorectal cancer risk and identifies a pleiotropic effect with inflammatory bowel disease — Supplementary Data 

# Variation at 2q35 (*PNKD* and *TMBIM1*) influences colorectal cancer risk and identifies a pleiotropic effect with inflammatory bowel disease

## Supplementary Data

files

- Supplementary Data - xlsx file
